# Increased Posterior Cingulate Functional Connectivity Following 6-Month High-Dose B-Vitamin Multivitamin Supplementation: A Randomized, Double-Blind, Placebo-Controlled Trial

**DOI:** 10.3389/fnut.2019.00156

**Published:** 2019-09-27

**Authors:** Luke A. Downey, Tamara N. Simpson, Talitha C. Ford, Grace McPhee, Chao Suo, Stephen P. Myers, Chris Oliver, Con K. K. Stough

**Affiliations:** ^1^Faculty of Health, Arts and Design, Centre for Human Psychopharmacology, Swinburne University of Technology, Melbourne, VIC, Australia; ^2^Institute for Breathing and Sleep, Austin Hospital, Melbourne, VIC, Australia; ^3^Cognitive Neuroscience Unit, Faculty of Heath, Deakin University, Melbourne, VIC, Australia; ^4^Brain and Mental Health Laboratory, School of Psychological Sciences, Monash Institute of Cognitive and Clinical Neuroscience, Monash University, Clayton, VIC, Australia; ^5^NatMed-Research, Division of Research, Southern Cross University, Lismore, NSW, Australia; ^6^National Centre for Naturopathic Medicine, Southern Cross University, Lismore, NSW, Australia; ^7^Oliver Nutrition, Pty Ltd, Lismore, NSW, Australia

**Keywords:** vitamin B, multivitamin, stress, resting state, functional connectivity, default mode network

## Abstract

B vitamins are essential for optimal brain and body function, and are particularly important for cortical metabolic processes that have downstream effects on mitigating oxidative stress. Oxidative stress has been linked to poor psychological outcomes including psychological distress, which has wide-reaching implications for the community and the workplace. Given work-related stress has been associated with poor mental health outcomes, high-dose B vitamin supplementation may be effective in improving brain function and psychological outcomes via attenuation of oxidative stress. This randomized, double-blind, placebo-controlled study investigated psychological outcomes following 6-month supplementation of a high-B-vitamin multivitamin in a large sample of healthy adults (*n* = 108, aged 30–70 years), as well as changes in default mode network functional connectivity in a subset of the original sample (*n* = 28). Improvements in occupational stress, general health, perceived stress, depressive symptoms, and mood profiles were identified for both active and placebo groups over time (*p* < 0.05 *corrected*). Seed-based functional connectivity analysis centered on the posterior cingulate cortex (PCC) showed that connectivity between the PCC and the caudate increased for the active treatment group, but decreased for the placebo group (*p* < 0.05 *corrected*). These findings reveal a substantial intervention effect for both active and placebo treatments, which could in part be associated with a placebo effect in subjective measures. There was, however, a significant treatment effect in the objective measure of functional connectivity, suggesting that reduced psychological stress and high-B-vitamin multivitamin supplementation may lead to an increase in DMN and caudate functional connectivity, which might reflect a strengthening of neurocircuitry within areas associated with reward and emotion at rest. Future studies should consider a placebo run-in methodology to reduce the placebo effect on the subjective measures of stress.

## Introduction

A diet rich in B vitamins is essential for optimal brain function, given their role in cortical metabolic processes that are essential for DNA synthesis, repair, and other methylation reactions within the central nervous system (1–3). The methylation process involved the conversion of the metabolite homocysteine to the essential amino acid methionine. Methionine is a precursor to *S*-adenosylmethionine (SAM), which is essential for the methylation of serotonin, dopamine and noradrenaline, which in turn play a central role in depressive symptoms (4). Higher concentrations of homocysteine thus suggests a disruption to the methionine cycle, which is a known risk factor for cortical inflammation and oxidative stress (1, 2), and has been linked to depressed mood (5) and deficits in cognitive performance (6). Several B vitamins are also integral to the synthesis of neurotransmitters critical to psychological wellbeing; for example, folate (vitamin B9) and vitamin B12 are required for single-carbon metabolism, which is involved in the synthesis and metabolism of serotonin and other monoamine neurotransmitters and catecholamines (7). Vitamin B6 is a cofactor for aromatic l-amino acid decarboxylase (AADC), an enzyme that catalyzes the decarboxylation of a variety of aromatic l-amino acids, which converts l-DOPA to dopamine and 5-HTP to serotonin (8) and regulates the levels of 5-HTP (9). Multi-vitamin supplementation more generally has also been associated with reduced perceived stress and anxiety (10) alongside reductions in homocysteine levels.

On the other hand, work-related stress has recently been recognized as a significant contributor to mental health issues including depression, putting additional strain on both the individual and the workplace, as well as the healthcare system at large (11–13). The Dunedin study, a 32-year longitudinal cohort study found that, for previously healthy young workers, work-related stress can lead to clinical levels of depression and anxiety (14). In fact, in Australia, “mental stress” made up 90% of mental disorder claims in 2015, costing $480AUD million (15); furthermore, workplace stress resulted in close to $14.81AUD billion lost in employee income in 2008 (16), and $3.1AUD billion lost in the Australian economy in 2012–13 (17). Given the emotional and financial cost of work-related stress, and the positive effects of vitamin B on mood, this study investigated the efficacy of 6-month high-B-vitamin multivitamin supplementation on psychological and mood outcomes, including work-related stress, as well as functional connectivity related to posterior cingulate based network.

Several studies have demonstrated a relationship between multivitamin supplementation with a predominance of B vitamins (B1, B2, B3, B5, B6, and B12) and improved mood, with as little as four-week supplementation shown to reduce self-reported stress, anxiety and tiredness (18–20), as well as depressive symptoms (21), while acute doses appear to increase individuals' feelings of contentedness (22). Natural plant extracts such as Bacopa and Pycnogenol have been shown to improve memory free recall and working memory (23–26) following 90 days of daily administration. Pycnogenol has also been shown to reduce concentrations of oxidative stress marker F2-isoprostanes (24), which has been associated with better episodic memory (27), while Bacopa has been shown to reduce anxiety (23). Furthermore, a 90-day intervention of high-dose B vitamin multivitamin was shown to be effective in reducing personal strain, depressive symptoms and confusion (28).

In the field of functional brain imaging, resting state functional magnetic resonance imaging (fMRI) is utilized to investigate interactive brain networks during passive rest, or in the absence of task demands (29). One major resting state network is the default mode network (DMN), which consists of the prefrontal cortex, posterior cingulate cortex (PCC), inferior parietal lobule, precuneus, hippocampal formation, and the temporal cortex. These regions are collectively involved in important internal cognitive functions, such as emotional processing, personal introspection, and autobiographical memory (30). Aberrant patterns of hyper-connectivity in resting state networks, particularly the DMN, have been associated with neuropsychiatric disorders, such as depression and schizophrenia (31–34), as well as prolonged psychological stress (35); although results are mixed (36). Increased functional connectivity between DMN regions, particularly in the cingulate gyrus, PCC, medial orbitofrontal cortex and precuneus, have also been associated with prolonged psychosocial stress in healthy adults (35). Such hyper-connectivity is particularly evident between the PCC and medial prefrontal cortex (35), which is in line with studies of depressed individuals, who also on average demonstrate increased connectivity between these regions (31–33).

Increased functional connectivity within the anterior components of the DMN in highly stressed individuals has been thought to reflect increased interaction between cortical regions involved in emotion processing and cognitive function, and a propensity for abnormal self-reflective thoughts (35). In contrast, increased connectivity between posterior DMN regions—the PCC and inferior parietal lobes—were thought to be more specific to prolonged emotional stimulus processing (35). Trait level stress has also been associated with increased functional connectivity between the left frontal superior gyrus and medial orbitofrontal cortex, middle cingulate gyrus, occipital middle regions, and in the right frontal middle gyrus, PCC and precuneus (37). The PCC is a central hub of the DMN, playing a role in internally directed cognition, as well as balancing externally directed thought and conscious awareness (38). Aberrant PCC function has been identified across psychiatric conditions such as depression, schizophrenia and autism, and has been further implicated in autobiographical memory retrieval (33, 38). The PCC has also been implicated in stress (39), with induced stress shown to increase connectivity between the PCC and caudate, and between the PCC, and thalamus and inferior parietal lobe (40). Subjective stress, on the other hand, has been more specifically associated with increased amygdala-hippocampal connectivity, not PCC connectivity (40). Given these patterns of aberrant DMN connectivity across both psychiatric conditions and elevated psychological stress and their connection with elevated levels of oxidative stress, examination of the potential moderation of these indices via alternative methods is required.

Psychological stress has a significant impact on the individual, the community, and their work-place. Given that perceived psychological stress has consistently been associated with increased functional connectivity of the DMN (35, 37, 40), coupled with evidence that B vitamins may aid in the reduction of psychological stress (18–20), it became important to tease out the possible interaction. This study investigates the extent to which high-B-vitamin multivitamin supplementation reduces functional connectivity to the PCC, which is the core of the DMN. We further investigate the efficacy of the high-B-vitamin multivitamin in reducing work-related stress, as well as secondary mood outcomes, such as depressive and anxiety symptoms. It was hypothesized that 6-month supplementation of a high-B-vitamin multivitamin would reduce subjective stress, improve mood and reduce functional connectivity.

## Materials and Methods

These data were collected as part of a large (*N* = 137) randomized, placebo-controlled, double-blind parallel groups design investigating the effects of Blackmores® Executive B Stress Formula on the primary outcome of work-related stress, as well as a range of secondary cognitive, stress, mood, health, personality, cardiovascular, biochemical, genetic, and neuroimaging outcomes (41). The study was registered with the Australian and New Zealand Clinical Trials Registry (ACTRN12613000294752), and was approved by the Swinburne University Research Ethics Committee (SUHREC 2012/293). The research was conducted in compliance with Good Clinical Practices (GCP) and in accordance with the guidelines of the Australian National Health and Medical Research Council and the Declaration of Helsinki (as revised in 2004). All participants provided written informed consent to participate in the study.

### Procedure

The intervention procedure of this study has been published previously (41, 42). Briefly, participants attended a screening visit to confirm study eligibility, with those eligible then randomized into placebo (*n* = 69) or active (*n* = 68) treatment conditions by random allocation using a computerized random number generator by a disinterested third party to ensure double-blinding; a subset of participants (*n* = 39) were enrolled to undergo an additional MRI scan protocol. Enrolled participants were provided their assigned treatment (detailed below in section Treatment). Participants then attended a baseline testing session and a 6-month post-treatment follow-up.

At the baseline and 6-month testing sessions, participants completed a food frequency questionnaire (FFQ) and psychometric measures, had blood samples taken for blood biomarkers [results in Ford et al. (42)], and resting state functional MRI where applicable (detailed below in section Whole sample psychometric statistical analysis Resting state functional MRI methods). Participants were contacted monthly to confirm treatment adherence and to record any significant life events or dietary changes. At the 6-month follow-up testing session, any remaining treatments were counted to confirm compliance with the treatment schedule.

### Treatment

Active and placebo treatments were in the form of large dark brown film-coated cream tablets, matched in color and size, and were manufactured by Blackmores® Australia. The doses of the active ingredients in each tablet are detailed in Supplementary Table 1, as well as the nutrients' percentage of recommended daily intake according to the National Health and Medical Research Council of Australia (NHMRC, 2018). All B vitamin doses were well above the recommended daily intake for adults aged 30–70 years, except biotin (vitamin B7, 66.7–80%) and folic acid (vitamin B9, 37.5%), confirming the supplement contained a high dose of B vitamins overall.

Participants were provided the treatment in unmarked bottles and instructed to take two tablets daily, one at breakfast and one at lunch, for the duration of 6 months. Each participant received enough tablets for 6 months, along with an additional week of tablets in case the post-treatment visit date was delayed, resulting in 350 tablets in total. To prevent any acute supplementation effects, participants were asked not to take any tablets on the day of their post-treatment testing session. Blackmores® Executive B Stress Formula is available over the counter. The placebo tablets contained a small amount of glucose and riboflavin (B2, 2 mg) to be matched for color and taste and to provide a similar urine coloration effect. No adverse events were reported as a result of the active or placebo treatment, or as a result of the study procedures.

### Participants

A total of 137 adults aged 30–65 years were enrolled in the randomized, double-blind, placebo-controlled study (female: *n* = 75, mean age [*SE*] = 44.1[1.1]; male: *n* = 62, mean age [*SE*] = 45.0 [1.4]). Of those enrolled, 130 participants completed the baseline visit (female: *n* = 73, mean age [*SE*] = 44.0[1.1]; male: *n* = 57, mean age [*SE*] = 44.6[1.4]), and received either the active (39 female, 27 male) or placebo (34 female, 30 male). Following the 6-month treatment intervention, 108 participants returned to complete the 6-month treatment and follow-up testing session of which 54 were in the active treatment group (female: *n* = 31, mean age [*SE*] = 44.6[1.57]; male: *n* = 23, mean age [*SE*] = 46.8[2.26]) and 54 were in the placebo group (female: *n* = 29, mean age [*SE*] = 42.6[1.97]; male: *n* = 25, mean age [*SE*] = 44.8[22.2]). Only those completing the 6-month intervention were included in the psychometric analyses. There were no sex differences in age for the final sample (*t* [*df* ] = −1.07[103], *p* = 0.285).

#### Resting State Functional MRI Participants

A subset of 39 participants were enrolled in the neuroimaging arm of the study. At baseline, 33 adults (female: *n* = 21, mean age [*SE*] = 41.1[2.1]; male: *n* = 12, mean age [*SE*] = 47.3[3.3]) underwent fMRI and received the active (10 female, 6 male) or placebo (11 female, 6 male) treatments. Following the 6-month treatment intervention, a total of 28 participants underwent the follow-up testing session, of which 14 were in the active treatment group (female: *n* = 8, mean age [*SE*] = 37.6[1.73]; male: *n* = 6, mean age [*SE*] = 49.3[4.90]) and 14 were in the placebo group (female: *n* = 8, mean age [*SE*] = 43.0[3.89]; male: *n* = 6, mean age [*SE*] = 47.0[6.43]). There were no group (*t* [*df* ] = −0.36(26), *p* = 0.725) or sex (*t* [*df* ] = −1.88(26), *p* = 0.072) differences in age for the neuroimaging sample. The functional MRI data were later checked for quality detailed in section Quality Check for fMRI images.

### Psychometric Measures

#### Occupational Stress Inventory—Revised (OSI-R)

The OSI-R is a 140-item measure of three dimensions of occupational adjustment: occupational stress (Occupational Roles Questionnaire, ORQ), psychological strain (Personal Strain Questionnaire, PSQ) and coping resources (Personal Resources Questionnaire, PRQ) (43). Items are rated on a 5-point Likert scale ranging from 1 (*rarely/never true*) to 5 (*true most of the time*). Higher scores on the QRQ and PSQ indicate high occupational stress and psychological stress and low scores on the PRQ indicate poor occupational adjustment. The OSI-R has been effective in identifying reduced personal strain and depressive symptoms following a 90-day B vitamin intervention (28).

#### Profile of Mood States (POMS)

The POMS is a 65-item measure of six mood dimensions: Anxiety, Confusion, Vigor, Anger, Depression, and Fatigue. A Total Mood Disturbance (TMD) score is computed as the sum of the subscales Anxiety, Confusion, Anger, Depression and Fatigue, minus Vigor. Responses are given on a 5-point Likert scale from 0 (*not at all*) to 4 (*extremely*) in relation to the degree to which participants identified with each of 65 mood-related adjectives within the past week. High scores indicate greater mood disturbance on all scales, except Vigor (44). The POMS has been effective in identifying reductions in tension-anxiety, anger-hostility, fatigue and confusion following a 90-day B vitamin intervention (28).

#### Perceived Stress Scale (PSS)

The PSS is a 10-item subjective measure of stress, assessing the degree to which respondents' life events are perceived as stressful. Responses are given on a 5-point Likert scale from 0 (*never*) to 5 (*very often*). Total scores range from 0 to 40 with higher scores indicating a greater degree of perceived stress and lower scores indicating effective coping (45). The PSS has been sensitive in detecting improvements in stress following 33 days of a high-dose B-vitamin and mineral supplement (20).

#### Beck Depression Inventory (BDI-II)

The BDI-II is a 21-item, self-report inventory designed to measure the severity of depressive symptoms (46). The BDI-II is one of the most widely used depression inventories in both clinical and research settings. Respondents indicate to what extent graded statements are true to their experience over the past 2 weeks, with statement scores ranging from 0 (no symptoms) to 3 (severe symptoms). Higher scores in the BDI-II indicate more severe depressive symptoms.

#### General Health Questionnaire (GHQ)

The GHQ comprises 28 items assessing changes in ones' ability to carry out normal daily functions, and the emergence of health and psychiatric symptoms over the past “few weeks” (47). Responses are given on a 4-point Likert scale ranging from 0 (*much less than usual*) to 3 (*much more than usual*). Total scores on the GHQ range from 0 to 84, with higher scores indicating more negative feelings or experiences and poorer general psychological health (48). The modified GHQ has been sensitive in detecting improvements in overall health following a 33-day high-dose B-vitamin and mineral intervention (20).

#### Food Frequency Questionnaire

Participants completed a food frequency questionnaire to quantify participants' consumption of different foods (e.g., fruits, vegetables, eggs, etc.). Participants rated how frequently they consumed each category on a 9-point scale ranging from 0 (Never) to 8 (More than 4 times a day). For their baseline assessment participants were required to indicate their average food frequency over the preceding 6-month period. For their follow-up assessment participants were required to indicate their average food frequency over the preceding 6-month period so as to track any changes in their diet that may have occurred over the course of the study.

#### Wechsler Abbreviated Scale of Intelligence (WASI)

The WASI (49) is an assessment of intelligence, and was administered by the researchers. All neuroimaging participants completed the vocabulary and matrix reasoning subsets of the WASI. The vocabulary subset is a 42-item task that requires participants to orally define words presented to them visually and orally. The matrix reasoning subtest requires participants to complete 35 incomplete grid patterns by pointing to, or stating, the correct pattern from five possible choices. The WASI is a reliable measure of intelligence for use in clinical and research settings (49).

### Whole Sample Psychometric Statistical Analysis

Log or square root transforms were applied to the whole sample psychometric data with a significant positive skew (skewness > 1): POMS Tension, Depression, Anger, Fatigue and Confusion, BDI-II, and GHQ. A series of linear mixed effects analyses with restricted maximum likelihood were conducted to investigate the effect of the 6-month high-B-vitamin multivitamin supplementation on work-related stress indices of occupational stress (ORQ) and psychological strain (PSQ), as well as POMS subscales, perceived stress (PSS), depressive traits (BDI-II), and general health (GHQ). Fixed effects were treatment group (active vs. placebo), time (baseline vs. 6 months), and the treatment × time interaction, with a random effect of subject entered into the model to account for within-subject variability. Where relevant, sex, age and IQ were entered as covariates due to sex differences in OSI-II PSQ, BDI-II, POMS Tension, POMS Vigor, and GHQ (*p* < 0.05), significant correlations between age and OSI-II OQR, OSI-II PSQ, BDI-II, POMS Anger, and PSS (*p* < 0.05). There were no relationships between years of education or IQ and psychometric measures, and no between-group differences in dietary intake (*p* > 0.05). All analyses were conducted using the Jamovi software (50) and the GAMLj package (51), and were corrected for false discovery rate (FDR) using the Benjamini and Hochberg method (52).

### Resting State Functional MRI Methods

For the subset of participants who underwent functional MRI, images were acquired using a 3T Siemens TIM Trio whole-body MRI system (Erlangen, Germany) with a 32-channel head coil equipped for echo planar imaging (EPI) at the Neuroimaging Facility located at Swinburne University of Technology. A T1 weighted sagittal localizing (T1) structural sequence was acquired for coregistration of the functional MRI data (176 slices, slice thickness = 1 mm, voxel size = 1.0 mm^3^, TR = 1,900 ms, TE = 2.52 ms, TI = 900 ms, bandwidth = 170 Hz/Px, flip angle = 9°, field of view = 256 mm, orientation sagittal). A gradient echo planar imaging sequence was employed to image resting state connectivity with eyes open and focused on a fixation cross (TE = 30 ms, TR = 2,500 ms, slices = 40, field of view = 224 mm, slice thickness = 3 mm, flip angle = 90°, 40 interleaved axial slices, bandwidth = 2,056 Hz/Px, matrix = 64 × 64). Participants pre-MRI anxiety levels were assessed immediately prior to the scan using the Spielberger's State Anxiety Inventory (53), so as to ensure that group differences in participants' scan-related anxiety did not affect potential group differences in functional connectivity between brain regions.

#### Quality Check for fMRI Images

MRIQC [v0.9.7; (54)] was used to check the fMRI image quality. Framewise displacement (FD) was used as the key parameter. For each scan, if the mean FD value was above 0.55 mm (55, 56), we excluded the scan from the further analysis, due to severe movement artifacts. Please refer to section Sub-study resting state functional MRI results for the final sample size for fMRI analysis.

#### Resting-State Functional MRI Preprocessing

Resting-state fMRI data were preprocessed using the Data Processing Assistant for Resting-State fMRI (DPARSF) toolbox [v4.2; (57)], which is based on the SPM software (Version 12, Welcome Trust Centre for Neuroimaging, London, UK) and the toolbox for Data Processing & Analysis of Brain Imaging (V4.1., DPABI; (58); http://rfmri.org/DPABI) using (59) (MathWorks Inc, Natick, MA). All processing in the study using SPM software was conducted using the same version (SPM12). In brief, preprocessing involved: (1) slice timing with the mid-slice as reference slice; (2) realignment of the 4D fMRI images; (3) normalization of the fMRI image to MNI space based on T1 structural image, including linear aligning fMRI image and T1 image, nonlinear warping the fMRI to MNI space by applying the warping between T1 image to MNI template, followed by resampling to 3 mm^3^ isotropic voxels; (4) removal of 9 nuisance regressors, including time course of CSF signal, white matter signal and global signal (CSF, WM and whole brain masks were selected from DPARSF standard masks), motion parameter from step 2 (moving parameters of six degree of freedom). Head motion scrubbing regressors (55, 60) were conducted during this step to further deal with movement artifacts (56), specifically, framewise displacement (FD) was calculated using a threshold set at 0.5 mm (default threshold in DPARSF). If FD of a volume was over this threshold, one volume before and two volumes after would be scrubbed. (5) smooth by 6 mm kernel; (6) detrending—removal linear global signal trends; and (7) band-pass filtering at 0.01~0.1 Hz.

#### Seed-Wise Functional Connectivity Analysis

The posterior cingulate cortex (PCC) seed was chosen for its role in the default mode network [DMN; (61)]. The PCC seed was generated based on prior research already reported (62), which was obtained by thresholding a typical DMN generated using Independent Component Analysis (ICA) toolbox (Group ICA Toolbox GIFT, http://mialab.mrn.org/software/gift/) from resting-state fMRI data across a group of similar participants. Individual DMN functional connectivity maps of the PCC seed were generated based on correlations between the mean signal time course within the seed region and the rest of the brain using the DPARSF toolbox. The raw functional connectivity maps were later z-transformed, before running the statistical analysis.

#### Resting-State Functional MRI Statistical Analysis

Voxel based statistical analysis of functional connectivity was conducted using SPM version 8 (Welcome Trust Centre for Neuroimaging, London, UK). The flexible factorial design included main effect of group (2 levels), main effect of time (2 levels), time × group interaction (4 levels) and four confounding covariates (age, sex, education years, and IQ), such design included all the validated images to maximize the degree of freedom. Contrast was set to test any significant time by group interaction after controlling for all covariates. An initial threshold was applied to define the cluster (*p*_uncorrected_ <0.001, K > 10), then the cluster with FDR corrected *p* < 0.05 was considered a significant region. *Post-hoc t*-tests were conducted on the values extracted from the Region of Interest (ROI) to further illustrate the direction of effect within groups. The functional connectivity values of significant regions were extracted to illustrate the direction of change. Spearman rank order (ρ) correlations were conducted to investigate the relationship between functional connectivity and work-related stress and psychological outcomes, corrected for FDR (52).

## Results

### Psychometric Results

Linear mixed models revealed no significant treatment effect for either of the work-related stress indices, or secondary psychometric measures (*p*s > 0.05); means and standard errors results are displayed in Table 1. Significant main effects for time (baseline vs. 6 months) were observed for OSI OQR (*B* = −3.38, *SE* = 0.96, *t*_(99.65)_ = −3.51, *p* < 0.001), OSI PSQ (*B* = −3.17, *SE* = 0.70, *t*_(96.61)_ = −4.53, *p* < 0.001), POMS Tension (*B* = −0.19, *SE* = 0.05, *t*_(103)_ = −4.19, *p* < 0.001), POMS Anger (*B* = −0.07, *SE* = 0.03, *t*_(79.46_) = −2.53, *p* = 0.013), POMS Fatigue (*B* = −0.29, *SE* = 0.06, *t*_(103)_ = −5.14, *p* < 0.001), POMS Confusion (*B* = −0.23, *SE* = 0.04, *t*_(103)_ = −5.96, *p* < 0.001), PSS (*B* = −1.75, *SE* = 0.28, *t*_(103)_ = −6.21, *p* < 0.001), BDI-II (*B* = −0.13, *SE* = 0.03, *t*_(70.28)_ = −4.86, *p* > 0.001), GHQ (*B* = −0.21, *SE* = 0.05, *t*_(1.3)_ = −4.07, *p* < 0.001) demonstrating a significant placebo effect. After controlling for FDR this effect of time remained significant for OSI OQR, OSI PSQ, POMS Tension, POMS Anger, POMS Fatigue, POMS Confusion, PSS, BDI-II and GHQ (*p* < 0.05 *corrected*).

**Table 1:** Means and standard error for psychometric indices between groups for the total sample at baseline and following 6-month supplementation of the high-B-vitamin multivitamin.

	**Baseline**		**6 Months**	
	**Active**	**Placebo**	**Active**	**Placebo**
*N*	54	54	54	54
OSI OQR	138.9 (2.71)	137.2 (3.55)	130.9 (3.26)	130.5 (3.51)
OSI PSQ	74.4 (2.5)	78.6 (2.57)	68.4 (1.88)	72.1 (2.72)
OSI PRQ	131.4 (2.54)	131.3 (2.77)	133.1 (3.09)	133.2 (3.14)
OSI Total	344.0 (4.39)	348.6 (5.12)	333.0 (4.29)	336.9 (4.68)
POMS Tension	6.9 (0.55)	7.2 (0.83)	4.9 (0.45)	5.2 (0.61)
POMS Depression	3.9 (0.52)	5.7 (1.11)	1.9 (0.36)	3.9 (0.87)
POMS Anger	4.8 (0.6)	7.4 (1.06)	3.1 (0.46)	4.5 (0.84)
POMS Vigor	16.4 (0.71)	15.8 (0.86)	16.5 (1.04)	16.3 (1.05)
POMS Fatigue	7.6 (0.78)	7.0 (0.82)	4.7 (0.56)	4.7 (0.67)
POMS Confusion	5.7 (0.5)	6.6 (0.68)	3.8 (0.38)	4.5 (0.46)
POMS TMD	14.3 (0.64)	15.2 (0.81)	10.5 (0.75)	12.0 (0.9)
PSS	5.0 (0.64)	6.9 (0.76)	1.5 (0.38)	3.2 (0.61)
BDI-II	18.6 (0.99)	19.3 (1.24)	16.5 (1.04)	14.7 (0.96)
GHQ	138.9 (2.71)	137.2 (3.55)	130.9 (3.26)	130.5 (3.51)

*OSI, Occupational Stress Inventory; OQR, Occupational Roles Questionnaire; PSQ, Personal Strain Questionnaire; PRQ, Personal Resources Questionnaire; POMS, Profile of Mood States; TMD, Total Mood Disturbance; PSS, Perceived Stress Scale; BDI-II, Beck Depression Inventory–Revised; GHQ, General Heath Questionnaire*.

### Resting State Functional MRI Sub-study

#### Sub-study Psychometric Results

For the subset of participants who underwent functional MRI, linear mixed models also revealed no significant treatment effect on work-related stress indices or secondary psychometric outcomes (*p*s >0.05; Table 2). Significant main effects for time (baseline vs. 6 months) were observed for OSI PSQ (*B* = −3.34, *SE* = 1.16, *t*_(24.3)_ = −2.90, *p* = 0.008), POMS Tension (*B* = −0.82, *SE* = 0.34, *t*_(26.0)_ = −2.40, *p* = 0.024), POMS Anger (*B* = −1.27, *SE* = 0.52, *t*_(26.0)_ = −2.42, *p* = 0.023), POMS Fatigue (*B* = −1.55, *SE* = 0.56, *t*_(26.0)_ = −2.77, *p* = 0.010), POMS Confusion (*B* = −1.07, *SE* = 0.37, *t*_(26.0)_ = −2.89, *p* = 0.008), PSS (*B* = −1.54, *SE* = 0.50, *t*_(26.0)_ = −3.08, *p* = 0.005), BDI-II (*B* = −1.93, *SE* = 0.48, *t*_(26.0)_ = −4.01, *p* < 0.001), GHQ (*B* = −1.71, *SE* = 0.68, *t*_(26.0)_ = −2.54, *p* = 0.017) demonstrating a significant placebo effect. After correcting for FDR, the effect of time remained significant for OSI PSQ, POMS Fatigue, POMS Confusion, PSS and BDI-II (*p* < 0.05 *corrected*).

**Table 2:** Means and standard error for psychometric indices between MRI participant groups at baseline and following 6-month supplementation of the high-B-vitamin multivitamin.

	**Baseline**		**6 Months**	
	**Active**	**Placebo**	**Active**	**Placebo**
*N*	14	14	14	14
OSI OQR	141.2 (6.67)	126.5 (6.18)	126.9 (6.44)	127.5 (6.22)
OSI PSQ	76.6 (6.52)	73.0 (5.96)	68.7 (4.49)	67.7 (4.19)
OSI PRQ	128.4 (6.31)	135.3 (5.27)	128.9 (7.39)	136.9 (4.86)
OSI Total	341.6 (11.03)	336.9 (9.47)	324.5 (8.68)	332.1 (9.18)
POMS Tension	6.9 (1.08)	5.4 (1.29)	5.3 (0.74)	3.7 (0.64)
POMS Depression	3.9 (0.79)	3.9 (1.19)	3.0 (0.94)	1.8 (0.74)
POMS Anger	4.4 (0.99)	6.9 (1.93)	3.9 (0.96)	2.2 (0.74)
POMS Vigor	16.1 (1.61)	18.6 (1.66)	17.1 (2.32)	18.9 (2.41)
POMS Fatigue	9.9 (1.87)	7.1 (2.0)	5.9 (0.58)	4.9 (1.53)
POMS Confusion	6.4 (1.35)	6.0 (1.24)	4.3 (0.71)	3.9 (0.82)
POMS TMD	15.2 (6.01)	10.7 (7.74)	5.3 (4.36)	−2.4 (5.93)
PSS	15.0 (1.56)	13.4 (1.30)	12.4 (1.97)	9.8 (1.46)
BDI-II	6.0 (1.68)	5.6 (1.49)	2.1 (1.17)	1.8 (0.76)
GHQ	20.2 (2.50)	16.3 (1.71)	18.1 (1.97)	11.5 (1.68)
SAI	31.4 (1.98)	27.1 (1.51)	27.1 (1.52)	28.7 (2.07)

*OSI, Occupational Stress Inventory; OQR, Occupational Roles Questionnaire; PSQ, Personal Strain Questionnaire; PRQ, Personal Resources Questionnaire; POMS, Profile of Mood States; TMD, Total Mood Disturbance, PSS, Perceived Stress Scale; BDI-II, Beck Depression Inventory–Revised; GHQ, General Heath Questionnaire; SAI, Spielberger State Anxiety Inventory*.

#### Sub-study Resting State Functional MRI Results

**Figure 1:**
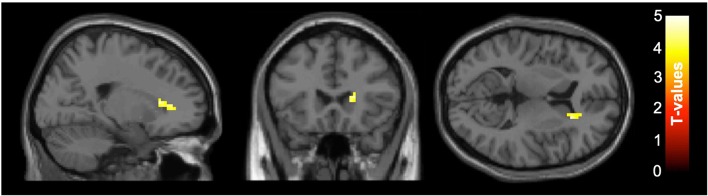
Axial slices of group ×time interaction for the functional connectivity between posterior cingulate cortex (PCC) seed and right caudate. After correcting for false discovery rate (FDR), functional connectivity of the PCC at rest increased in the right caudate (*k* = 60, *p*_FDRcorrected_ =0.046, *t* = 4.14 [21 24 9]) for the active group compared to the placebo group between baseline and follow-up. The color-bar indicates the *t*-value, left side is subject's left side. Slice numbers on the top left of each image represent the standard MNI coordinates in axial view.

**Figure 2:**
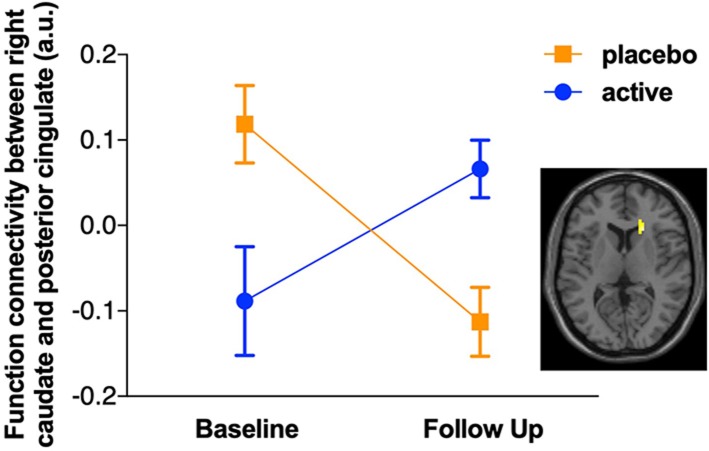
Plot of the group × time interaction for functional connectivity between posterior cingulate cortex (PCC) seed and right caudate. After correcting for false discovery rate (FDR), a significant group × time interaction was observed for the functional connectivity between posterior cingulate cortex (PCC) seed and right caudate (*k* = 60, *p*_FDRcorrected_ = 0.046, *t* = 4.14 [21 24 9]). The *post-hoc t*-test suggested an increase of functional connectivity for the active group (*t* = 2.06, *p* = 0.050), while there was a significant decrease for the placebo group (*t* = −3.52, *p* = 0.002).

For the subset of functional MRI data after quality control, five scans were excluded due to motion artifacts (three participants' baseline scans and one participant's scans at both baseline and follow-up), resulting total sample size of *N* = 56, baseline/follow-up = 29/27. The intervention group showed increased functional connectivity between the PCC and the right caudate from baseline to follow up compared with the control group (see Figure 1). A group × time interaction showed this cluster was significant at the right caudate (*k* = 60, *p*_FDRcorrected_ = 0.046, *t* = 4.14 [21 24 9]). To illustrate the different change between the groups, the functional connectivity values were extracted and are plotted in Figure 1, with the group × time interaction shown in Figure 2. The *post-hoc t*-test suggested an increase of functional connectivity for active group (*p* = 0.050, *t* = 2.06), while there's a significant decrease for placebo group (*p* = 0.002, *t* = −3.52). We also performed the same imaging statistical analysis using only paired samples (*N* = 50, baseline/follow-up 25/25) and the group x time interaction showed the cluster remained at the right caudate (*k* = 91, *p*_FDRcorrected_ = 0.006, *t* = 5.62). Although the active treatment group was slightly more anxious than the placebo group at baseline (see Table 2), there was no significant relationship between pre-scan state anxiety and functional connectivity (*r* = −0.06, *p* = 0.696) or state anxiety and functional connectivity change across the 6-month intervention (*r* = 0.19, *p* = 0.182).

#### Correlation Between Resting State Functional Connectivity and Psychological Outcomes

Spearman ρ correlations were conducted to investigate the relationship between PCC-right caudate functional connectivity, and work-related stress and psychological outcomes at baseline and follow-up separately. There were no significant correlations between functional connectivity and psychometric outcomes at baseline or at the 6-month follow-up (*p* > 0.05).

## Discussion

This study was the first randomized, double-blind, placebo-controlled trial investigating the extent to which 6-month supplementation with a high-B-vitamin multivitamin would affect work-related stress and PCC seed functional connectivity, in addition to psychological and mood outcomes. Although the supplement showed no additional improvement in work-related stress, or psychological and mood outcomes, there was an effect on functional connectivity between the right caudate and PCC, the core of the DMN. An area of interest of the DMN was the PCC, given the hyper-connectivity within this region that is associated with poor psychological outcomes (35, 37, 40), and the well documented reduction in stress, anxiety and depressive symptoms following vitamin supplementation (18–22, 28). The 6-month supplementation increased functional connectivity between the PCC and right caudate for the active treatment group, while there was a significant decrease in functional connectivity between these regions for the placebo group. In terms of psychological and mood outcomes, both active and placebo treatment groups showed improvement across almost all measures. Although the implications of these findings should not be overstated, the data suggests that poor mood is associated with aberrant PCC-caudate connectivity, while the increase in connectivity following high-B-vitamin multivitamin supplementation might be associated with concurrent reduction in connectivity due to reduced stress, and strengthened connectivity of positive reward-cognition hubs.

While stress is considered a normal psychological and physiological response to external stressors, the coping strategies employed by individuals in dealing with stress is a key factor in overcoming the perceived challenges they face (63). Within the workplace, when stressors continue beyond a manageable level, individuals are at risk of developing psychological and physiological disorders that are likely to impact negatively on work productivity due to absenteeism and work dysfunction (64). Poor productivity and absenteeism are costly for both the individual, the workplace, and government bodies (16, 17). Multivitamin supplementation has been shown to improve mood, and reduce stress, anxiety and depressive symptoms (18–21), while B vitamin supplementation specifically has been shown to reduce personal strain, depressive symptoms, and confusion (28). In line with these findings, improvements in mood, psychological, and workplace stress outcomes occurred following high-B-vitamin multivitamin supplementation; however, despite the rigorous randomized, placebo-controlled, double-blind parallel groups design of the study, a similar improvement was evident for the placebo treatment group, indicating a notable placebo effect. A likely explanation for such an improvement across both treatment groups is an intrinsic motivation within participants to improve their negative mood status, and manage workplace stress more effectively.

Although more recent studies demonstrate increased DMN connectivity for those experiencing a high degree of stress (35, 37), decreased resting state connectivity between the PCC and caudate has also been identified in people with early depression (36). In contrast, reduced functional connectivity of the DMN, ventral attention network, and sensorimotor network has been identified in those who have recovered from chronic stress (65). Soares et al. (65) also report normalized deactivation in the DMN, sensorimotor network, and auditory network following recovery from chronic stress, suggesting that the effect of chronic stress on specific resting state networks can be recovered through targeted intervention.

The caudate is part of the striatum, which is involved in reward processing and in turn, is a key driver of pleasure and motivation (66, 67). The increase in connectivity between the caudate and PCC observed following high-B-vitamin multivitamin supplementation may, therefore, reflect a strengthening of neurocircuitry within brain regions that are associated with reward and emotion (36). Increased functional connectivity between intra- and inter-hemispheric frontal and temporal regions has also been associated with higher blood levels of vitamin B1 and B6 (68). In this large cohort study, higher blood vitamin B6 levels were associated with increased functional connectivity between the left insula and middle temporal gyrus, and the left inferior frontal gyrus and right middle frontal gyrus; while connectivity was reduced between left inferior frontal gyrus and supramarginal gyrus. Increases in connectivity associated with B1 levels were also identified inter-hemispherically, between the left middle temporal gyrus and right superior frontal regions, left frontal and right occipital regions, and left and right supramarginal gyrus. Intra-hemispheric connectivity, however, was generally reduced between anterior and posterior regions, suggesting functional reorganization due to aging (68). The authors investigated connectivity between seed regions where there was significant cortical atrophy associated with vitamin B levels, thus do not specifically investigate resting state networks. Nevertheless, these findings suggest that higher vitamin B levels are associated with regionally specific increases in functional connectivity. Increased plasma vitamin B and D have also been associated with reduced DMN and fronto-parietal network efficiency—the extent to which there is high local connectivity, and short average path length between regions—which could suggest stronger long-range connections within those networks (69). It is noteworthy that the multivitamin supplementation in this study included a small concentration of choline, which is modulates cell signaling processes and membrane structure (70). Choline and folate metabolism in the brain are closely linked, and B vitamin + choline supplementation has been shown to reduce plasma homocysteine and promote neuroplasticity, improving sensorimotor functioning following stroke (71). In line with these findings, increased functional connectivity between the PCC and right caudate in combination with increased B-vitamin levels, suggests a strengthening of neurocircuitry and concurrent improvements in psychological outcomes following high-B-vitamin multivitamin supplementation.

Although the current findings suggest that 6-month high-B-vitamin multivitamin supplementation may increase connectivity between the PCC and right caudate, the improvements in psychological outcomes for the placebo group might themselves explain the reduction in aberrant functional connectivity observed for those who were not taking the multivitamin. As per the above discussion, improving psychiatric outcomes has been associated with reduced functional connectivity (65), which may explain the reduced functional connectivity observed for the placebo group given their significant improvement in workplace stress, depressive traits and anxiety. Furthermore, these differences in functional connectivity were independent of MRI scan-related state anxiety; however, it is important to acknowledge that baseline functional connectivity between the PCC and right caudate was significantly higher for the placebo compared to the active treatment group, despite no group differences in demographic characteristics or psychological measures. Baseline differences might, therefore, be related to alternate demographic or clinical factors, such as lack of sensitivity of the measures used in this study. Nevertheless, the repeated measures nature of this study allows for the effect of the 6-month intervention on functional connectivity to be examined, whilst accounting for individual differences and group differences at baseline. It is indeed possible, that any reduction in functional connectivity due to improved psychological outcomes for the active treatment group may have been masked by a strengthening of neurocircuitry between the positive reward-cognition hubs due to vitamin B supplementation. This interaction might result in a net increase in functional connectivity between the PCC and the right caudate following supplementation.

These data reveal a notable reduction in workplace stress, anxiety, depressive traits, and additional mood outcomes that are attributable to both the active and placebo treatments. These reductions were in combination with changes in functional connectivity between the PCC in the DMN and the caudate nucleus of the basal ganglia. Together, these findings suggest that poor mood is associated with aberrant PCC-caudate connectivity, while high-B-vitamin multivitamin supplementation for 6 months may strengthen positive functional connectivity. Despite these findings, it is important to note that the final functional connectivity sample was relatively small (*n* = 14 per group), and that the supplement contained several additional vitamins and minerals (e.g., vitamin C, vitamin E; see Supplementary Table 1). These additional ingredients, however, are considered less likely to have contributed to the change in functional connectivity. It is also important to note that fortification of folate is common in several Westernized countries, including Australia, which may contribute to the lack of research reporting positive effects of folate supplementation in otherwise healthy populations. Despite these limitations, the findings provide preliminary evidence for the effect of B vitamin supplementation on functional connectivity from DMN nodes, and suggest that the intersection of reduced psychological stress and high-B-vitamin multivitamin supplementation may lead to an increase in DMN and caudate functional connectivity.

Given the large placebo effect, future studies should employ a different methodology to the current study where a placebo run-in is used. The current results do not allow us to make any conclusions about the subjective effects of B Vitamins on stress given the large placebo effect. In this regard the subjective component (i.e., self-reporting of questionnaire data) may be best considered as a failed trial with future research needing to employ a placebo run-in in order to minimize the placebo effect. This would then allow a more rigorous assessment of whether B vitamins improve subjective work stress. Nevertheless, this study adopted a randomized, double-blind, placebo-controlled methodology, the results of which make a significant and novel contribution to the scientific literature regarding the impact of high-dose B vitamin supplementation on psychological stress and brain functioning, and may have significant implications of improving work-related stress.

## Data Availability Statement

The datasets generated for this study are available on request to the corresponding author.

## Ethics Statement

This study was carried out in accordance with the recommendations of guidelines of the Australian National Health and Medical Research Council with written informed consent from all subjects. All subjects gave written informed consent in accordance with the Declaration of Helsinki. The protocol was approved by the Swinburne University Research Ethics Committee.

## Author Contributions

LD, CKS, SM, and CO: conceptualization. LD, CKS, and TS: methodology. CS: software. TS, LD, and TF: validation. LD, CS, and TF: formal analysis. TS, GM, and SM: investigation. CO: resources. GM and LD: data curation. TF, CS, TS, and LD: writing—original draft preparation. TF, TS, and CS: visualization. CKS, LD, and CS: supervision. LD, SM, and TS: project administration. CKS and LD: funding acquisition. All authors: writing—review and editing.

### Conflict of Interest

CO was employed by Oliver Nutrition, Pty. Ltd. The remaining authors declare that the research was conducted in the absence of any commercial or financial relationships that could be construed as a potential conflict of interest. The funders had no role in the design of the study; in the collection, analyses, or interpretation of data; in the writing of the manuscript; or in the decision to publish the results.
