# Supplementary material for: Increased Posterior Cingulate Functional Connectivity Following 6-Month High-Dose B-Vitamin Multivitamin Supplementation: A Randomized, Double-Blind, Placebo-Controlled Trial
Source: Front Nutr. 2019 Sep 27;6:156. doi: 10.3389/fnut.2019.00156 (PMC6776972; doi:10.3389/fnut.2019.00156)
Supplement: Supplementary file 1 [file Table_1.DOCX]

Supplementary Material

**Supplementary Table 1.** Dose and percent of recommended daily intake (RDI) for the active ingredients in Blackmores^®^ Executive B Formula.

| **Active Ingredients (per tablet)** | **Dosage** | **RDI/AI** | |
| --- | --- | --- | --- |
|  |  | **Male** | **Female** |
| Vitamin B1 (thiamine) | 75 mg | 6,250% | 6,818% |
| Vitamin B2 (riboflavin) | 10 mg | 769% | 909% |
| Vitamin B3 (nicotinamide/niacin) | 100 mg | 625% | 714% |
| Vitamin B5 | 68.7 mg | 1,145% | 1,718% |
| Vitamin B6 (pyridoxine) | 25 mg | 1,923% * | 1,923% * |
|  |  | 1,470% ^#^ | 1,667% ^#^ |
| Vitamin B7 (biotin) | 20 μg | 66.7% | 80% |
| Vitamin B9 (folic acid) | 150 μg | 37.5% | 37.5% |
| Vitamin B12 (cyanocobalamin) | 30 μg | 1,250% | 1,250% |
| Calcium phosphate | 100 mg | 10% | 10% |
| Calcium ascorbate | 145 mg | 14.5% | 14.5% * |
|  |  |  | 11.2% ^#^ |
| Ascorbic acid | 130 mg | 289% | 289% |
| Total vitamin C | 250 mg | 556% | 556% |
| d-alpha-tocopheryl acid succinate (vitamin E) | 41.3 mg | 413% | 590% |
| Magnesium phosphate | 140 mg | 33.3% | 43.8% |
| Potassium phosphate monobasic | 117.3 mg | 3.1% | 4.1% |
| Choline birartrate | 25 mg | 4.5% | 5.9% |
| Lecithin | 50 mg | NR | NR |
| Inositol | 25 mg | NR | NR |
| Avena sativa (oats) | 100 mg | NR | NR |
| Passifloraincarnata (passion flower) | 250 mg | NR | NR |

Note: RDI or Adequate Intake (AI) is given according to the Nutrient Reference Values for Australia and New Zealand (NHMRC, 2018). Recommendations are for the age range of 30–70 years, * = 31–50 years, ^#^ = 51–70 years. NR = not reported.

**Reference**

National Health and Medical Research Council (NHMRC), Nutrient Reference Values for Australia and New Zealand Including Recommended Dietary Intakes; Available online: https://nhmrc.gov.au/sites/default/files/images/nutrient-refererence-dietary-intakes.pdf (accessed on 18 Oct 2018)
